# A single-subject research design evaluating a co-created yoga program for adults with gynecologic cancer: feasibility study protocol

**DOI:** 10.1186/s40814-023-01435-7

**Published:** 2024-01-16

**Authors:** Jenson Price, Jennifer Brunet

**Affiliations:** 1 School of Human Kinetics, University of Ottawa, 125 University Private, Montpetit Hall, Room 339, Ottawa, ON K1N 6N5 Canada; 2 Cancer Therapeutic Program, Ottawa Hospital Research Institute, The Ottawa Hospital, Ottawa, ON Canada; 3 Institut du Savoir Montfort, Hôpital Montfort, Ottawa, ON Canada

**Keywords:** Mind–body, Oncology, Single case, Community

## Abstract

**Background:**

Worldwide, > 1.3 million adults are diagnosed with a gynecologic cancer each year, affecting their wellbeing and quality of life. This manuscript describes the protocol for a study that sought to assess the feasibility, acceptability, and fidelity of a community-based co-created yoga program and proposed evaluative methods, and estimate program effects on self-reported outcomes.

**Methods:**

Using a multiple baseline single-subject research design with a follow-up phase (ABA), quantitative and qualitative data were collected from program participants and the instructor. Participants were randomly assigned to varying baseline lengths and completed weekly surveys for 3–5 weeks pre-program. Then, participants engaged in a bi-modal 12-week hatha yoga program consisting of 2 60-min group classes a week, with optional supplemental features (January–April, 2023). Participants completed surveys after classes 1, 12, and 24. All yoga classes were audio- and video-recorded. Post-program, participants completed surveys 1, 4, and 8 weeks after the last class and took part in a semi-structured interview 1 week after to discuss program acceptability, suitability, relevance, and potential benefits. Feasibility outcomes (i.e., recruitment, retention, and program adherence rates, engagement with optional program features) were tracked by the instructor assistant and study team during the study. The yoga instructor was interviewed about their experience delivering the program 2 weeks after the last class.

**Planned analysis:**

Feasibility outcomes will be analyzed using descriptive statistics. Interview transcripts will be coded using reflexive thematic analysis. Class recordings will be coded using duration and frequency coding. Survey responses for self-reported outcomes will be analyzed visually and using multilevel modeling.

**Expected outcomes:**

Data will help determine refinements, if any, required to the program and instructor guidebook, implementation approach, and proposed evaluation methods before scale-up projects and definitive trials are started.

**Trial registration:**

ClinicalTrials.gov NCT05610982. November 3, 2022.

**Supplementary Information:**

The online version contains supplementary material available at 10.1186/s40814-023-01435-7.

## Background

Adults diagnosed with a gynecologic cancer report physical [1, 2] and psychosocial [3–6] side effects associated with their cancer and its treatment, which can have a lasting impact on their quality of life (QoL) [7–10]. Physical activity programs have been identified as means of supporting QoL after cancer treatment due to potential improvements in physical and psychological outcomes [11]. Often though, these adults report low levels of physical activity [12] (e.g., any voluntary bodily movement, encompassing all activities at any intensity) and note that treatment side effects can reduce their ability to participate in physical activity [13]. Many have therefore sought to establish that yoga, a mind–body practice, can be a form of physical activity that can be done by persons with physical limitations and confers physical and psychosocial benefits during and after cancer treatment [14–17]. A meta-synthesis of 24 articles indicates yoga helps women manage the adverse side effects of cancer and its treatments, rediscover strength and physical abilities, embrace a positive outlook and relationship with themselves, develop strategies for coping with stressors, foster social connections and support, and become more attentive and mindful [18]. Yet, offering yoga programs without involving end-users (i.e., those who might be interested in, or affected by, research findings) in the whole research process (i.e., from conception to evaluation) can lead to suboptimal engagement and user experience [19]. Involving end-users in the whole research process is beneficial to help identify and prioritize user-relevant topics for the research agenda and research questions, offer pragmatic criticism on the extent to which the research is relevant or appropriate for users across each phase of the research, and can lead to programs that meet the needs and wants of participants [20].

In 2021, a formal inter-disciplinary partnership between 2 researchers, a psychologist at The Ottawa Hospital, and a representative from a community organization serving people diagnosed with cancer and their families (the Ottawa Regional Cancer Foundation; ORCF) was established to co-create a contextually relevant and suitable yoga program. The partners collaborated with adults diagnosed with gynecologic cancer and yoga instructors to involve end-users and elicit their feedback in the development of the yoga program and a guidebook to assist instructors in delivering the program as intended (Price J, Praamsma N, Harris C, Brunet J: Co-producing a yoga program for adults diagnosed with gynecologic cancer: a consensus study, submitted). The co-creation process resulted in a 12-week bi-modal program featuring 2 weekly 60-min Hatha yoga classes to a group of 5 to 7 participants, with 3 additional optional features participants can engage with (organized social time, journaling, and pre-recorded videos for at-home practice). Though a definitive trial will be warranted to assess the direct and indirect effects of the yoga program on adults diagnosed with gynecologic cancer, it is not indicated at this time [21]; rather, it is first necessary to establish feasibility, acceptability, and fidelity of the program and evaluative methods.

There are several ways to assess these parameters. Feasibility involves evaluation of (a) recruitment capability and sample characteristics, (b) data collection procedures and measures, (c) acceptability and suitability of a program and study methods, (d) resources and ability to manage and implement a program and study, and (e) preliminary data on participants’ responses to a program [22]. Acceptability involves evaluation of the extent to which people delivering or receiving a program consider it to be appropriate based on anticipated or experienced cognitive and affective responses to a program [23]. Last, fidelity involves evaluation of the extent to which people delivering a program under study implement it with accuracy and conformity [24]. It is necessary to establish these parameters to ensure study outcomes are due to participation in the program and not due to other factors (e.g., high dropout, biased sample, lack of reliable and valid outcome assessments, poor program adherence, compliance, or fidelity).

Although there are multiple study designs for evaluating feasibility, acceptability, and fidelity of programs and evaluative methods, the trend has been to use feasibility or pilot trials [25, 26]. Pilot- or feasibility randomized controlled trials may not be the most appropriate or cost-effective choice when exploring the suitability of an untested group-based yoga program in a community setting [27]. Without knowledge of end-users’ interest and engagement in the program, it is not possible to estimate with confidence the number of *eligible* individuals that a program will have, meaning that the sample size required for a randomized controlled trial may not be met. As well, randomized controlled trials are vulnerable to many types of bias (e.g., selection bias, ascertainment bias, observer bias). Single-subject research design (SSRD) studies have been offered as valuable for assessing feasibility, acceptability, and fidelity while minimizing needed resources and implementation time [28–32]. Moreover, SSRDs offer a rigorous approach to understanding individual variability when examining causal relationships between a program and outcomes in clinical and applied settings [29, 31, 32].

## Purpose and study objectives

The purpose of this article is to describe the protocol (Version 1, November 3, 2022) for a feasibility study delivering a group-based yoga program to adults diagnosed with gynecologic cancer using a bi-modal format (in-person and online), and aiming to: (1) assess trial methods and program feasibility, (2) assess trial methods and program acceptability, (3) assess researcher and yoga instructor fidelity to trial methods and program protocol, and (4) estimate the effects of the program on end-user prioritized self-report outcomes. The practice of publishing protocols is growing owing to a lack of high-quality reporting in articles detailing yoga interventions and programs [18]. Protocol publication also makes replication easier, promotes transparency, enhances awareness of the trial to avoid duplication efforts, and serves as reference for forthcoming publications of results [33].

## Methods/design

This manuscript was written in accordance with the SPIRIT (Standard Protocol Items: Recommendations for Interventional Trials) guidelines ([34]; see Additional file 1).  At the time of writing this protocol baseline data collection has been completed and follow up is about to begin. Trial results will be reported according to the Consolidated Standards of Reporting Trials (CONSORT) 2010 statement for N-of-1 trials [35] and the 21-item Checklist Standardising the Reporting of Interventions For Yoga (CLARIFY) reporting guidelines [36]. Any modifications to the study methods or program will be reported with the results.

## Trial design

This was a SSRD study (unblinded), employing a multiple baseline approach with a follow-up phase (i.e., ABA design; [32, 37, 38]), wherein quantitative and qualitative data were collected from people receiving the program (i.e., adults diagnosed with gynecologic cancer; participants) and those delivering it (i.e., yoga instructor). In this design, “A” refers to a baseline (control) or non-intervention (follow-up) phase, and the “B” refers to the intervention phase wherein participants participate in the program. The key to this design was that participants were randomly assigned to 1 of 3 baseline lengths, varying from 3 to 5 weeks following guidelines [38], wherein they completed 3 to 5 weekly surveys online correspondingly. As minimal change was anticipated during this observation A phase, assessments were weekly to minimize phase length. Then, the yoga program was introduced to all participants and participants completed surveys online at the start (week 1), mid- (week 6), and end-of (week 12) program to observe trends in outcomes during the program. After the program ended, participants completed surveys online 1-, 4-, and 8-weeks post-program to continue observing trends in outcomes post-program. To maximize research participation from enrollment to study completion while considering possible latency effects, the follow-up period was 8 weeks.

## Participants, program, and outcomes

### Eligibility criteria

Inclusion criteria were as follows: (1) at least 18 years of age, (2) received a diagnosis of non-metastatic gynecologic cancer, (3) able to read, speak and understand English, (4) access to the Internet and an audio-visual device (e.g., computer, smart phone), and (5) able and willing to travel to the ORCF (Ottawa, Ontario, Canada) twice a week for 2 weeks at the start of the yoga program. Exclusion criteria were: (1) being non-ambulatory (i.e., unable to walk or require the assistance of a mobility device), and (2) currently practice yoga at least once a week or have practiced consistently in the last 6 months (i.e., once a week for 8 weeks).

### Yoga program and study setting

The approach and methods (i.e., consensus panel meeting, focus groups) used to co-create the yoga program with end-users are presented elsewhere (Price J, Praamsma N, Harris C, Brunet J: Co-producing a yoga program for adults diagnosed with gynecologic cancer: a consensus study, submitted). Version 1 of the yoga program was 12 weeks and offered 2 weekly 60-min group-based Hatha-style classes (a total of 24 classes) and up to 10 participants could enrol in the program. For this study, 2 programs were offered concurrently at different times of day (i.e., 9:30–11:00am and 7:00–8:30pm). Two weeks prior to the first group yoga class, the yoga instructor asked participants to complete a brief 1-page intake form and participate in a 1-on-1 online consultation for approximately 15 min via Zoom to review the intake form and discuss any physical concerns or limitations. The purpose of the intake form and meeting was to ensure the safety of participants by assisting the instructor to better tailor the program to suit each participants’ concerns and limitations. It also served to familiarize participants with Zoom and offer technological assistance, if needed. Weeks 1 and 2, participants had to attend classes in-person. Starting week 3 through to week 12, the program became bi-modal such that participants could choose to attend the classes in-person or live online via Zoom. Participants were required to enter a password to attend online classes to prevent unauthorized persons from joining. The in-person classes were offered in a spacious room at the ORCF located in the basement. The room had windows to let in natural light, wooden floors, high ceilings, and ceiling lighting, it was void of mirrors, and it was located in close proximity to non-gendered washrooms. The ORCF lent participants chairs, blocks, bolsters, yoga blankets, and yoga straps during in-person classes. The research team offered participants a yoga mat donated by Lululemon at the start of the first class to use during in-person classes and at-home for the duration of the program (i.e., they could take it home with them); they were asked to return it after the last class for re-use in future studies.

#### Class aims and content

The safety and wellbeing of participants was at the forefront of the program, such that the elements of the practice could be tailored to suit the needs of each participant so as to make it inclusive and accessible. Participants were encouraged to connect movement with breath as they engaged in dynamic movement, engaged multiple muscle groups as they moved through a series of postures, and regulated their breathing and heart rate through breath practices and meditation. To promote program fidelity, the instructor was given the Instructor Guidebook (Version 2), which contained guidance on gynecologic cancer, inclusive language and behaviors, program and class structure, and a flexible and adaptable base class plan for the instructor to use. Each class included moving, stretching, and balancing through a series of poses (asanas), awareness of breath (breathwork; pranayama), and cultivating the connection between mind and body (meditation; dhyana and dharana) [15, 39]. During the yoga sequences, introspection was central—that is, participants were encouraged to focus on their bodies' activities and the internal sensations of their bodies. Breathwork provided a foundation for the calming of the mind, and meditation encouraged them to observe their present-moment experiences with openness, acceptance, and nonjudgment [40–44]. See Table 1 for an overview of the class layout.

**Table 1:** Overview of class layout for the yoga program for adults diagnosed with gynecologic cancer

Activity	Description	Timing
Arrival	The instructor seeks to make the class environment welcoming and connect with participants as they transition into the class	5 min
Warm-up	Starts with a breath practice, followed by a structured sequence of movements from a seated position intended to help participants center themselves in the present, get their heart elevated, and prepare their mind and body to fully engage in the class by tuning into their body, heart rate, breathing, and energy level	15 min
Sequence 1	A structured sequence of standing movements intended to help participants connect their movement with their breath as they engage in dynamic movement	10 min
Sequence 2	A structured sequence of standing and balancing movements intended to help participants connect their movement with their breath and engage multiple muscle groups as they flow through a series of shapes	15 min
Restoration	A structured sequence of movements to help participants regulate their breathing and heart rate and begin the calming process and transition into a meditation practice	20 min
Social time	The instructor prompts conversation among participants intended to promote reflection and connection	5–10 min
Departure	When participants conclude their social time or the scheduled class time ends, the instructor transitions participants out of the class by thanking them for attending and inviting any individual questions	5 min

#### Supplemental features

Based on end-user input, the program also included organized group discussions, journaling, and pre-recorded videos that participants could engage with, if they chose. For the former, participants could remain in the ORCF or in the Zoom meeting at the conclusion of the class to socialize with the other participants and ask the instructor questions to allow participants to develop feelings of relatedness and belongness while sharing with others. The yoga instructor initiated this by announcing the beginning of the group discussion and asking participants to share, if they wanted, but she was instructed to not facilitate conversations; however, the instructor deviated from the protocol and engaged in conversation to facilitate conversation with online participants (when necessary). For journaling, participants received a journal and pen during the first class to use as they saw fit to record self-reflections, express their thoughts, and release emotions that may have surfaced during the class. The instructor encouraged participants to bring their journal to each class and write in it at the end of class. Finally, starting week 3, participants had access to an online database of 10 pre-recorded short-duration yoga practices (10–15 min) with videos for breathwork, meditation, warm-up, main sequences, and restoration to support their at-home practice. The pre-recorded classes allowed participants to self-select the intensity, frequency, and duration of the practice to meet their needs in-between classes guided by the instructor.

#### Instructor and instructor assistants

A certified yoga instructor, with experience working with clinical populations, was hired and trained. The yoga instructor was supported by 2 volunteer undergraduate students in Human Kinetics (1 per program) to ensure participants attending in-person or online were receiving the necessary support required for a safe and enjoyable experience. Given their pivotal role, the yoga instructor and instructor assistants completed a 3-h training class facilitated by JP. Training topics and practical activities included observations, feedback on delivering a mock class, relevant information on gynecologic cancer treatments and side effects, discussions on inclusive and supportive language and behaviors such as using inviting, accepting, and welcoming language, offering choices, avoiding qualifiers, empowering introspection, tailoring according to participants’ needs, seeking consent, and encouraging participants to meet their bodies where it was. Ongoing supervision was provided; it consisted of 30***-***min team meetings every 2 weeks to discuss participants’ progress, challenges, tailoring efforts, and feedback on class notes, as well as hear about the yoga instructor and instructor assistants’ experiences delivering/supporting the delivery of classes. During the meetings, participants' engagement and adverse events were reviewed to ensure participants’ safety because a data monitoring committee was not needed for the yoga program due to the intervention being non-invasive with minimal risk of harm.

### Participant timeline

Study duration varied between 23 and 25 weeks, depending on baseline length. Recruitment took place from November 25, 2022 to December 22, 2022, with JP screening potential participants by phone to ensure eligibility criteria was met. Informed verbal consent was obtained from interested and eligible participants during the call to reduce burden of coming in-person prior to online data collection; they were emailed a copy of the consent form for their records. Then, 6 weeks prior to the start of the program (December 23, 2022), participants were randomized by JP into a 3-, 4-, or 5-week baseline length for A phase, in a 1:1:1 ratio using the Clinical Trial Randomization Tool offered by the National Cancer Institute (https://ctrandomization.cancer.gov/). Starting December 26, 2023, on a weekly basis, they received an email (at around the approximate same day/time to encourage equal spacing in-between assessments) with a link to a secure site to complete an online survey for 3, 4, or 5 weeks (depending on baseline length allocation). Once baseline A phase ended, the program B phase began (January 31, 2023). Participants took part in the 12-week yoga program and were asked to complete a survey package after the first class (week 1), after the 12th class (i.e., mid-point of program; week 6), and after the 24th class (i.e., end of program; week 12). With participants’ consent, classes were audio- and video-recorded to track participants’ engagement in the classes and the instructor’s fidelity to the program. For the follow-up A phase (started April 27, 2023), participants were asked to complete a survey online 1, 4, and 8 weeks after the last class. For all phases, participants were asked to complete surveys within 48 h of receiving the link. All participants (regardless of adherence to the program) were asked to complete all program B phase and follow-up A phase surveys. Participants were invited to take part in an acceptability interview 1 week after their last class. Interviews took place virtually via Microsoft Teams. The yoga instructor was invited to take part in an interview via Microsoft Teams approximately 2 weeks after the last class. See Table 2 for the schedule of assessments. The CONSORT flow diagram will be completed and presented in a forthcoming publication to summarize the process of recruitment and follow-up of participants within the study (see Additional file 2).

**Table 2:** Schedule of assessments

	Throughout study	Pre-data collection	Baseline					Program			Follow-up			Program end
Measures	Nov 25, 2022–Jun 16, 2023	Nov 25–Dec 23, 2022	Dec 26, 2022–Jan 23, 2023					Jan 31–Apr 20, 2023			Apr 27–Jun 16, 2023			May 1–May 12, 2023
			− *t*_5_^a^	− *t*_4_^a^	− *t*_3_	− *t*_2_	− *t*_1_	*t*_1_	*t*_2_	*t*_3_	*t*_4_	*t*_5_	*t*_6_	
Eligibility screen		X												
Informed consent		X												
Baseline length allocation (3, 4, or 5 weeks)		X												
Feasibility metrics	X													
Participant interview														X
Instructor interview														X
Fidelity monitoring								X^b^	X^b^	X^b^				
Primary definitive trial outcomes			X^a^	X^a^	X	X	X	X	X	X	X	X	X	
Secondary definitive trial outcomes			X^a^	X^a^	X	X	X	X	X	X	X	X	X	
Sociodemographic and medical characteristics			X^a^	X^a^	X^a^									
Adverse events								X^b^	X^b^	X^b^				

*Notes*. ^a^Depending on group allocation. ^b^Tracked continuously throughout the program

### Sample size

A conventional power calculation was inappropriate for this feasibility study given the novel nature of delivering the program at the ORCF [45, 46]; rather, the target sample size was 20 participants based on end-user recommendations and pragmatic considerations. During the co-creation process, end-users recommended that class size be capped at 7 participants per program/group to allow the instructor to provide adequate feedback to participants, which was increased to 10 per program to account for up to 30% attrition based on the research team’s previous experience delivering group-based physical activity programs. In addition, end-users recommended running programs in the morning and evening to accommodate varying scheduling needs (e.g., energy levels, work). Pragmatically, due to the group-based nature of the program, the novelty of the program, and the context of the study (i.e., Covid-19-related shifts in lifestyles, priorities, and preferences), it was necessary to establish recruitment and enrolment rates as well as acceptability of novel program features prior to committing resources to run multiple iterations of the program. Thus, a SSRD was chosen as the study design wherein an appropriate sample size is generally small (e.g., range from 1 to 13 [32, 47]). Repeated assessments helped to ensure there were a sufficient number of data points to perform statistical analyses with sufficient power [32]. See Feasibility Outcomes for pertinent confidence intervals (CI).

### Recruitment

Adults diagnosed with gynecologic cancer were recruited via: (1) healthcare provider referral, (2) registry mailout, and (3) posters and word of mouth. For recruitment strategy 1, a psychologist at The Ottawa Hospital asked her colleagues to inform potentially eligible patients about the study and share contact information for those interested in learning more to JP. For recruitment strategy 2, adults diagnosed with gynecologic cancer who provided consent to be contacted for research purposes at The Ottawa Hospital were contacted by mail and invited to participate (and thus a Data Use Agreement was established between the University of Ottawa and The Ottawa Hospital). Interested individuals self-referred by contacting JP. For recruitment strategy 3, recruitment posters and brochures were placed at the ORCF and shared via their newsletter and website. Finally, individuals enroled in the study were encouraged to share JP’s contact information with people whom they believed could be interested in participating.

### Outcomes

#### Primary: Feasibility [throughout trial]

Feasibility outcomes were as follows: (1) *recruitment rate*, (2) *study retention rate*, (3) *program adherence rate*, and (4) *participant engagement.* (1), (2), and (4) were tracked by JP; (3) was tracked by the yoga instructor assistants. Recruitment rate was measured by recording the number of individuals who consented to participate in the study out of those who were assessed for eligibility. Retention rate was measured by recording how many participants completed assessments, with the primary endpoint as the final assessment at week 20 (i.e., 8 weeks post-program) to provide valuable data about whether participants are willing to continue data collection after the program for future large-scale trials. Adherence rate was measured by recording the number of classes participants attended (out of 24) regardless of modality (i.e., in-person or online), though a breakdown of modality use will be presented. Participant engagement was measured by recording how many of the 24 group discussions participants took part in (including attending vs actively participating by engaging in discussion), how many journal entries participants’ self-reported completing, and how many pre-recorded practices participants self-reported watching.

Based on recent yoga programs with cancer survivors [16, 48–50], targets were set a priori. Feasibility of the study methods and program will be deemed if: (a) recruitment: > 50% of adults who were assessed for eligibility consent to participate (CI = 34.5–65.5%), (b) retention: final follow-up assessment at week 20 completed by ≥ 75% of the sample (CI = 56–94%), (c) adherence: ≥ 75% of the 24 yoga classes completed (CI = 70.7–79.3%), and (d) participant engagement: (1) 50% of participants attended the optional group discussion (CI 28.1–71.9%), and of those, 50% spoke at least once during the discussion (CI 19.0–81.0%), (2) 50% of participants completed ≥ 1 journal entry per week (CI 28.1–71.9%), and (3) 50% of participants watched ≥ 1 pre-recorded video per week for at least 50% of the time they had access to the videos (5 weeks) (CI 28.1–71.9%).

#### Primary: Acceptability—Participants [1-week post-program completion]

All participants were invited to complete an in-person or virtual semi-structured interview with JP 1 week after the last class (May 1, 2023). Acceptability of the study methods and program were assessed. Interviews included closed (i.e., quantitative) and open-ended (i.e., qualitative) questions about participants’ experiences, thoughts, and perspectives on: (1) relevance of the program overall and its specific features, (2) suitability of the program overall and its specific features, (3) perceived benefits of the program overall and its specific features, (4) problems/concerns experienced during the program, and (5) suitability and problems/concerns with trial methods.

#### Primary: Acceptability—Yoga Instructor [at program cessation]

Within 2 weeks of teaching their last class, the yoga instructor took part in an audio-recorded semi-structured interview to explore their experience delivering the program, as well as their thoughts about the content of the program and guidebook, delivery and content of training, ongoing supervision, and whether they felt confident that they had enough knowledge to teach the yoga program as intended. As well, they were asked to share any difficulties encountered while delivering the program, offer suggested amendments, and their views of fidelity to the guidebook. Their responses will be taken into account when reflecting on if/what changes/modifications should be considered to optimize the program and facilitate its implementation.

#### Primary: Fidelity [throughout trial]

The classes were recorded and will be used to assess the instructor’s fidelity to the program protocol, including: (1) layout of the class (i.e., timing and sequencing of class phases aligns with the program protocol), and (2) interactions with participants (i.e., instructor engages with the participants in manner that aligns with pre-specified preferred behaviors identified in the instructor guidebook). Instructor fidelity will be measured using duration and frequency coding to compare recorded yoga classes to the program protocol. Fidelity will be considered if: (1) layout of the class: ≥75% of phases were the prescribed length and ≥75% of class phases were in the prescribed order, and (2) interactions with participants: ≥75% of instructor behaviors were preferred behaviors.

### Effectiveness outcomes to inform future trial

Several patient-reported outcomes and putative mechanisms of change were identified based on end-user feedback and literature. In turn, participants were asked to complete pertinent questionnaires to provide preliminary data on the benefits of the program and inform measurement decisions for a future definitive trial. All of the questionnaires and assessments have been used and/or validated previously with cancer survivors. They were chosen on the basis of empirical evidence showing yoga can affect these outcomes, theories, and end-users and partners’ input. The study measures were collected at the time points listed below using SurveyMonkey.

#### Primary—patient-reported outcomes [baseline A phase, program B phase, follow-up A phase]

Patient-reported outcomes were assessed using the following questionnaires: Functional Assessment of Cancer Therapy-General (QoL; [51]), Perceived Cognitive Abilities sub-scale of the Functional Assessment of Cancer Therapy—Cognitive Function (cognitive functioning; [52]), Functional Assessment of Chronic Illness Therapy—Fatigue Scale (fatigue; [53]), Female Sexual Distress Scale-Revised modified for 7-day recall (feelings and problems regarding sexuality; [54]), Body Image Scale modified for 7-day recall (body image; [55]), and Perceived Stress Scale modified for 7-day recall (perceived stress; [56]).

#### Secondary—putative mechanisms of psychosocial outcomes [baseline A phase, program B phase, follow-up A phase]

Testing mechanisms of change will be central to understand how the program works in a future definitive trial. Thus, several theoretically grounded outcomes were assessed at each timepoint using the following measures to ascertain feasibility of data collection: Acceptance and Action Questionnaire II modified for 7-day recall (psychological flexibility; [57]), Integrated Self-Discrepancy Index (self-discrepancy; [58]), and Experiences of Embodiment Scale modified for 7-day recall (embodiment; [59]).

### Additional outcomes

#### Personal and medical factors [baseline A phase; single timepoint]

Participants self-reported sociodemographic (age, gender identity, ethnicity, civil status, work/education status, income, and comorbidities), medical information (height, weight, cancer type and stage, type and protocol of treatments received for their cancer [i.e., surgery, chemotherapy, radiation, immunotherapy, hormonal]), and perceived physical and mental health (via 5-point scale) within the first survey only.

#### Adverse events [throughout the trial]

Becoming more physically active, through yoga, is very safe for most adults diagnosed with cancer and can yield many health benefits [18, 60, 61]. Though the risk of injury was estimated to be very low, the yoga instructor educated participants on the warning signs that may indicate a problem as per Canadian Society for Exercise Physiology guidelines (e.g., chest discomfort, unusual shortness of breath, dizziness or light-headedness, heart rhythm abnormalities) and told participants to seek immediate medical attention should 1 of these signs occur. Adverse events (i.e., any unfavorable and unintended sign, symptoms, or disease) definitely, probably, or possibly related to engaging in yoga were tracked. To this end, any adverse events occurring during participation in the study were to be documented by the yoga instructor. Each class, the yoga instructor recorded adverse event shared by participants (if any) in their notes and reported them immediately to JP via email. At the time of reporting to JP, the program was to be paused for the participant with the adverse event until clearance from an appropriate healthcare provider to resume the program was obtained. Any reported adverse events were to be shared with Institutional Review Boards and will be reported in forthcoming publication(s) of study results. Beyond participating in the yoga program, there was a risk that participants experience distress in response to certain questions included in the online survey and/or posed during the interviews; participants received a list of free resources to consult in case this occured during the consent process (e.g., Canadian Cancer Society–Peer support / cancer information specialist: 1-888-939-3333).

### Data management and analysis

Quantitative data will be downloaded from SurveyMonkey, imported into Statistical Package for the Social Sciences (SPSS) for analysis, cleaned, and subjected to quality checks. Descriptive statistics will be computed to describe the sample at baseline and to report on feasibility and acceptability outcomes. Categorical variables will be described using proportions and frequencies, and continuous variables with means and standard deviations (or with medians and interquartile ranges for data that are not normally distributed). CIs will be reported along with point estimates for each outcome.

Audio recordings from qualitative interviews will be transcribed verbatim, imported into NVivo for analysis, and coded using a hybrid approach of deductive and inductive reflexive thematic analysis [62].

Additional analyses (i.e., descriptive analyses, visual inspection) will be performed to describe overall levels and the extent to which patient-reported outcomes and putative mechanisms of psychosocial outcomes (i.e., anticipated primary and secondary definitive trial outcomes, respectively) change. Additionally, although exploratory, multilevel modeling will be used to estimate change in primary and secondary outcomes. Restricted Maximum Likelihood will be used instead of Full Maximum Likelihood because Restricted Maximum Likelihood has been shown to lead to better estimates with smaller sample sizes. Models will be estimated with no Level-2 covariates to maximize power. This said, effect sizes (e.g., measures of variance explained, measures of standardized effect size) will be reported for primary and secondary outcomes as this was a feasibility study that was not designed to test the efficacy of yoga. Because there are a number of ways to approach multilevel modeling and each model tested proceeds by estimating and comparing a series of unconditional and conditional models, full technical details and estimation methods will be described when reporting results.

Finally, once the main analyses are conducted to address the study aims, exploratory analyses may be undertaken to address subsequent research aims that will make unique theoretical and empirical contributions.

## Ethics and dissemination

### Research ethics approval and protocol amendments

Ethics approval for the yoga program protocol was granted by the Ottawa Health Science Network Research Ethics Board on September 27, 2022 (file no.: 20220544-01H) and by the University of Ottawa’s Office of Research Ethics and Integrity on November 11, 2022 (file no.: H-10–22-8671). The trial was registered with the ClinicalTrials.gov database (no.: NCT05610982) on November 3, 2022. Any important protocol modifications (e.g., changes to eligibility criteria, recruitment procedures) will be reported promptly to relevant parties (e.g., team members, Institutional Review Boards, trial registry) and will be reported in forthcoming publication(s).

### Consent and confidentiality

Verbal informed consent was obtained from participants over the phone prior to data collection, and the yoga instructor prior to completing the interview. All data (i.e., interview recordings/transcripts, survey responses) are considered confidential and electronic files containing personal information can only be accessed by the investigators and study staff who sign a confidentiality form. A unique code is produced for each participant and used on all corresponding documentation and files to ensure anonymity. Furthermore, personal information that may enable participants to be identified will be removed from interview transcripts upon transcription.

### Access to data

Data sharing is restricted. No data will be deposited within public data repositories as participants and the yoga instructor were assured that their data would be kept private and confidential to the extent permitted by law, and that only the research team would have access to their data. De-identified electronic files containing quantitative and qualitative data are only to be shared with the research team for analysis. All quantitative and qualitative electronic data files are password-protected and stored on password-protected computers/laptops, a shared drive/server (i.e., The Ottawa Hospital server), and a web-based secure and encrypted data storage service (i.e., SurveyMonkey’s Canadian Data Centre); files will be kept for at least 5 years and possibly longer. Paper materials will be stored for 5 years following the completion of the study in locked cabinets in locked offices whose access is limited, after which point these will be destroyed securely (i.e., shredding).

### Dissemination plans

Study results will be shared with academic audiences via presentations at scientific meetings and publications in peer-reviewed journals. Results will be submitted to ClinicalTrials.gov no later than 1 year after study completion. To facilitate communication of results to non-academic audiences, presentations will be made to the ORCF board of directors and results will be presented in a publicly available report on the ORCF website. In addition, results will be shared via social media and a video summarizing the trial will be posted on the corresponding author’s website. As well, yoga instructors interested in using the instructor guidebook will be able to request access through the corresponding author’s website.

## Discussion

Yoga is a promising form of physical activity that can support psychosocial outcomes for women diagnosed with cancer when practiced regularly and safely [18]. Yet, most programs have some form of disconnect between program characteristics and participants’ needs [19]; thus, highlighting the need to co-create programs with end-users (i.e., yoga instructors, adults diagnosed with a gynecologic cancer). Prior to a definitive trial to assess the effects of the program and effort to scale up implementation, it was necessary to assess the feasibility, acceptability, and fidelity of the study methods and program as well as potential benefits of the program to ensure viability and value.

## Deliverables and implications

The results of the study described in this article will provide valuable knowledge and insight to refine the current program, materials to facilitate its delivery as planned (i.e., instructor guidebook), and the methods used to evaluate it. Further, data on patient-reported outcomes and putative mechanisms of change will support the design of a sufficiently powered future trial. In the long term, if successful, the deliverable of this study will be a yoga program suitable for delivery in a community setting. This program will aim to provide yoga instructors and community organizations with a resource for adults diagnosed with a gynecologic cancer to support bio-psycho-social outcomes related to QoL.

### Supplementary Information

**Additional file 1. **SPIRIT 2013 Checklist: Recommended items to address in a clinical trial protocol and related documents.

**Additional file 2. **CONSORT Flow Diagram.

## Data Availability

Not applicable.

## Abbreviations

QoL: Quality of life
ORCF: Ottawa Regional Cancer Foundation
SSRD: Single-subject research design
SPIRIT: Standard Protocol Items: Recommendations for Interventional Trials
CONSORT: Consolidated Standards of Reporting Trials
CLARIFY: Checklist Standardising the Reporting of Interventions For Yoga
SPSS: Statistical Package for the Social Sciences
