# Supplementary material for: A single-subject research design evaluating a co-created yoga program for adults with gynecologic cancer: feasibility study protocol
Source: Pilot Feasibility Stud. 2024 Jan 16;10:8. doi: 10.1186/s40814-023-01435-7 (PMC10790429; doi:10.1186/s40814-023-01435-7)
Supplement: Supplementary file 2 — Additional file 2. CONSORT Flow Diagram. [file 40814_2023_1435_MOESM2_ESM.docx]

Figure 1. CONSORT Flow Diagram

**Analysis**

Analyzed (*n*= )

Excluded from analysis (*n*= )

**Follow-up Phase**

**Week 18-25**

Follow-up (*n*= )

Completed assessment 4 (*n*= )

Completed assessment 5 (*n*= )

Completed assessment 6 (*n*= )

**Program Phase**

**Week 6-17**

Yoga Program (*n*= )

Completed assessment 1 (*n*= )

Completed assessment 2 (*n*= )

Completed assessment 3 (*n*= )

**Baseline**

**Week 1-5**

Allocated to 3-week baseline phase (*n*= )

Completed assessment -t_3_ (*n*= )

Completed assessment -t_2_ (*n*= )

Completed assessment -t_1_ (*n*= )

Allocated to 4-week baseline phase (*n*= )

Completed assessment -t_4_ (*n*= )

Completed assessment -t_3_ (*n*= )

Completed assessment -t_2_ (*n*= )

Completed assessment -t_1_ (*n*= )

Allocated to 5-week baseline phase (*n*= )

Completed assessment -t_5_ (*n*= )

Completed assessment -t_4_ (*n*= )

Completed assessment -t_3_ (*n*= )

Completed assessment -t_2_ (*n*= )

Completed assessment -t_1_ (*n*= )

Baseline Phase allocation (*n*= )

Referred by healthcare provider (*n*= )

Self-referred (*n*= )

**Enrollment**

Excluded (*n*= )

- Not meeting eligibility criteria (*n*= )
- Declined to participate (*n*= )
- Other reasons (*n*= )

Assessed for eligibility (*n*= )
